# Left-Sided Bochdalek's Hernia in a Young Adult: A Case Report and Literature Review

**DOI:** 10.1055/s-0041-1731443

**Published:** 2021-07-19

**Authors:** Mark Portelli, Mark Bugeja, Charles Cini

**Affiliations:** 1 Department of Surgery, Mater Dei Hospital, Msida, Malta

**Keywords:** Bochdalek's hernia, gastric volvulus, congenital abnormalities, Gore-Tex mesh, dysphagia

## Abstract

**Purpose** Bochdalek's hernia is a type of congenital diaphragmatic hernia occurring secondary to a defect in the posterior attachment of diaphragm. This condition commonly presents with respiratory insufficiency in infants. To date, there are less than 100 cases of Bochdalek's hernia presenting in adults published in the literature. The mainstay treatment of Bochdalek's hernia involves reduction of hernial contents back into the peritoneal cavity with a tensionless graft repair closing the diaphragmatic defect.

**Case Presentation** We present an atypical case of the Bochdalek hernia presenting in a previously healthy 16-year-old male who presented to the Accident and Emergency department with a 2-day history of dysphagia and loss of breath. The Bochdalek hernia was confirmed on computed tomography (CT) imaging and the patient underwent surgical repair with Gore-Tex mesh.

**Conclusion** The report shows a rare case of the Bochdalek hernia in a young adult, successfully managed with a laparotomy.

Bochdalek's hernia is a type of congenital diaphragmatic hernia occurring secondary to a defect in the posterior attachment of diaphragm. It constitutes 85% of cases. 1 The term was first coined in 1848 by Vincent Alexander Bochdalek. 2 3 It commonly presents in prenatal and postnatal patients and to date, less than 100 cases of this type of hernia are documented in the literature as presenting in adults. Overall, 80 to 90% of Bochdalek's herniae are commonly found on the left. 2

We present a case of a left-sided Bochdalek's hernia in a 16-year-old male who was successfully treated. We believe that Bochdalek's hernia being rare in adults can be easily misdiagnosed and should be considered in patients presenting with gastrointestinal and respiratory symptoms. As a result, we believe this case report is a significant addition to the current literature.

## Case Report

We present a case of a 16-year-old boy with no significant past medical history who presented with a 2-day history of decreased appetite and dysphagia. He also complained of epigastric pain. He did not experience any fevers, chills, rigors, or hematemesis and experienced one episode of shortness of breath 2 days prior to admission.

**Fig. 1:**
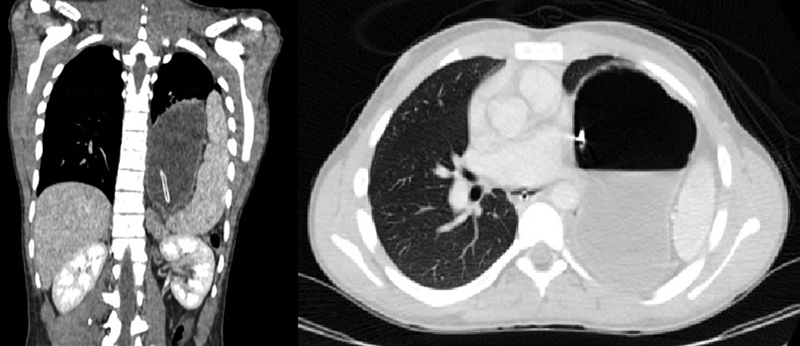
Coronal and axial view of contrast-enhanced computed tomography.

A chest X-ray followed by a contrast-enhanced computed tomography (CT) scan of the thorax ( Fig. 1 ) were performed in view of minimal improvement after analgesia. These showed a distended stomach together with an enlarged spleen on the left side of the chest with shift of the mediastinal structures to the right side. The findings were suggestive of the Bochdalek hernia, with obstruction of the herniated part of the stomach probably due to volvulus.

**Fig. 2:**
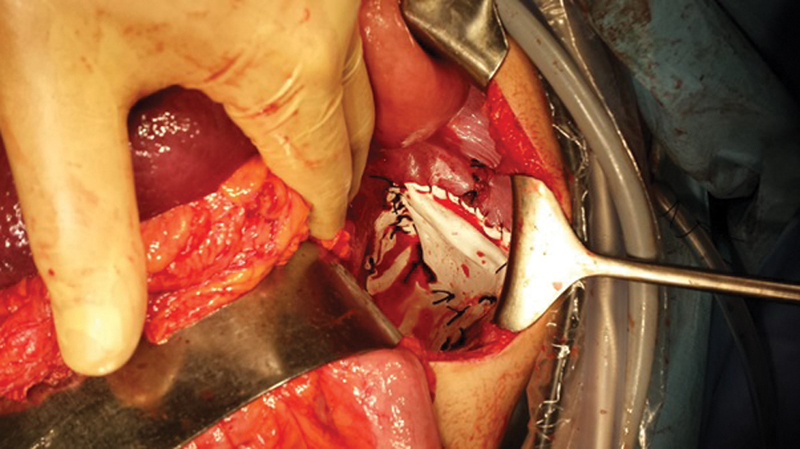
The hernia was reduced successfully and application of Gore-Tex mesh to the diaphragmatic defect.

He subsequently underwent a laparoscopic repair initially which was converted to laparotomy. A large congenital left diaphragmatic hernia was observed with an acutely torted intrathoracic spleen and stomach, as well was left part of transverse colon, and small bowel loops. Despite an attempt to perform a laparoscopic repair, the surgery had to be converted to an open procedure. The hernia was reduced successfully and a Gore-Tex mesh was applied to the diaphragmatic defect, approximately 7 cm in diameter and secured with prolene sutures ( Fig. 2 ). In view of pulmonary hypoplasia and pneumothorax, a chest drain was left in situ.

The patient had an uneventful recovery and was discharged 10 days after the procedure.

## Discussion

Bochdalek's hernia is a subtype of diaphragmatic hernia, through the posterolateral foramen of Bochdalek. 4 The term was coined by a Czech Anatomist, Vincenz Alexander Bochdalek, in 1848. 3 5 Overall, 80 to 90% of Bochdalek's herniae commonly present on the left side. 4 6 Right-sided herniae are rare (10% of cases), as the right pleuroperitoneal canal closes earlier and the liver buttresses the right diaphragm. 2 7 8 Complete separation of the thoracic and abdominal cavities occurs by the eighth week of gestation. 6 The hernial defect is congenital in origin due to failed closure of the pleuroperitoneal ducts, affecting 1 in 2,200 to 2,500 live births with a male to female ratio of 3:1. 6 8 Despite being commonly diagnosed prenatally or in the immediate postnatal period, diagnosis can be late in 5 to 25% of cases and commonly present with gastrointestinal and respiratory problems. 4

Presentation in adults commonly occurs due to delayed rupture of peritoneal sac which contains the viscera or plugging of the diaphragmatic defect by a solid organ and subsequent herniation after increased intra-abdominal pressure such as in obesity and pregnancy. 1 4 6 9

Since the hernia commonly results in pulmonary hypoplasia and therefore pulmonary symptoms, it is commonly diagnosed in neonates and infants. 5 8 With the advent of easy access to X-ray and CT imaging, it is considered a very rare occurrence in adults. 4 To date, there are less than 100 reports published in the literature on Bochdalek's hernia presenting in adults. 2 5

Symptomatic presentation varies between children and adults. In children, the commonest presentation is that of respiratory distress. Adults tend to present with more chronic symptoms such as pleural effusion, chest pain, dyspnea, abdominal pain, dysphagia, and postprandial fullness. 2 5 In 25% of cases, this can be fully incidental in nature and identified on a routine chest X-ray. 4 5 Patients may also present with chronic symptoms such as recurrent lower respiratory tract infections and chest pain. 5 In our case, the patient presented with epigastric pain, decreased appetite, and dysphagia.

The contents of the hernial sac varies and depends on the size of the herniation. In half of acute presentations, the sac contains colon. In 40% of cases the sac can contain other viscera notably the stomach, liver, gall bladder, small bowel, and spleen. Right-sided herniae commonly include liver and intestine. Left-sided herniae include spleen, small intestine, stomach, and colon. 4 6 8 Mortality and morbidity is significantly increased in cases where patients present with incarceration and strangulation of abdominal viscera. 8

In the absence of trauma, chest X-ray readily diagnoses the presence of Bochdalek's hernia usually heralded by the presence of loops of bowel or soft tissue within the chest. 2 However, the findings on plain X-ray can be easily confused for pulmonary sequestration, pulmonary lobar collapse or consolidation. 6 Due to the broad differential diagnosis from a two-dimensional image on a chest X-ray, contrast-enhanced chest CT is commonly employed. This allows a more accurate understanding and characterization of the diaphragmatic defects. The gold-standard for diagnosis of Bochdalek's hernia remains a double-contrast axial CT. 2 In cases where CT scan is not performed, Bochdalek's hernia can be misdiagnosed in up to 38% as pleural effusion, empyema, or pneumothorax. 8

The current mainstay of treatment remains surgical repair due to risk of strangulation and visceral herniation. 2 6 7 8 Two procedures, namely, the transthroacic and transabodminal approaches have been described in the literature. The transthoracic approach commonly through a lateral thoracotomy is considered relatively easier in comparison to the transabdominal approach. 6 Laparoscopic repair is nowadays the gold standard of treatment for Bochdalek's hernia owing to the relatively lower complication rate and an average hospital stay of 4 days. 4 6 During the procedure, contents are reduced, and the diaphragmatic defect is closed using nonabsorbable sutures and a synthetic mesh. Other surgical meshes, such as biological or synthetic biodegradable meshes have been used in previous accounts. However, currently no data shows superiority of one type of mesh over the others. 6 The rate of recurrence is relatively low. 8 Despite surgery being the mainstay of treatment, anatomic abnormalities can vary from case to case and therefore surgical treatment needs to be individualized based on findings on CT imaging. 5 In this case, the patient underwent a conversion of a laparoscopic procedure to laparotomy as reduction of hernial contents proved difficult using the laparoscopic method.

If left unrepaired, previously reported cases have documented complications such as gastric gangrene, ischemic bowel necrosis, respiratory failure, splenic infarction, and gastric volvulus. 6

## Conclusion

The case presented in this report illustrates the pathway employed in the typical diagnosis in an emergency setting and the associated management of Bochdalek's hernia. This report presents only one example of clinical presentation and therefore is not generalizable to all cases of Bochdalek's hernia. However, the management remains common in all documented cases in the literature.

The presence of congenital diaphragmatic hernia in adults remains rare and misleading even for experienced clinicians as the condition will mimic other diseases. Timely diagnosis and a high index of clinical suspicion are required to avoid complications. Surgical repair is recommended for all cases of Bochdalek's hernia.
